# Prevalence of metabolic syndrome among primary and secondary school teachers: a systematic review and meta-analysis

**DOI:** 10.3389/fpubh.2026.1913303

**Published:** 2026-08-17

**Authors:** Tuillang Yuing-Farías, Pablo Hernández-Araya, Sudip Datta Banik, Francisco José Berral-de la Rosa, Pablo A. Lizana

**Affiliations:** 1 Facultad de Salud, Escuela de Kinesiología, Universidad Santo Tomás, Viña del Mar, Chile; 2 Programa de Doctorado en Ciencias de la Actividad Física y del Deporte, Universidad Pablo de Olavide de Sevilla, Sevilla, Spain; 3 Exercise and Rehabilitation Sciences Institute, School of Physical Therapy, Faculty of Rehabilitation Sciences, Universidad Andrés Bello, Santiago, Chile; 4 Laboratory of Biometry and Nutrition, Department of Human Ecology, Centro de Investigación y de Estudios Avanzados del Instituto Politécnico Nacional (CINVESTAV), Mérida, Yucatán, Mexico; 5 Centro de Investigación en Rendimiento Físico y Deportivo (CIRFD), Universidad Pablo de Olavide de Sevilla, Sevilla, Spain; 6 Laboratory of Epidemiology and Morphological Sciences, Instituto de Biología, Pontificia Universidad Católica de Valparaíso, Valparaíso, Chile; 7 Center for Interdisciplinary Research in Biomedicine, Biotechnology and Well-Being (CID3B), Pontificia Universidad Católica de Valparaíso, Valparaíso, Chile

**Keywords:** cardiometabolic risk, meta-analysis, metabolic syndrome, occupational health, systematic review, teachers

## Abstract

**Background:**

Schoolteachers are considered at increased risk for metabolic syndrome due to high psychosocial demands, sedentary occupational routines, and restricted opportunities for regular physical activity. However, the prevalence of metabolic syndrome among primary and secondary schoolteachers has not been systematically quantified.

**Objective:**

To synthesize and quantify the available evidence on the prevalence of metabolic syndrome in primary and secondary school teachers worldwide through a systematic review and meta-analysis.

**Methods:**

A systematic review and meta-analysis were conducted in accordance with the PRISMA 2020 guidelines and the protocol was prospectively registered in PROSPERO (CRD420251034493). Searches were performed in the PubMed, Scopus, and Web of Science databases from their inception until April 27, 2026. Observational studies reporting the prevalence of MetS in primary and secondary school teachers were included. Methodological quality was assessed using the National Heart, Lung, and Blood Institute’s tool for cohort and cross-sectional observational studies. Prevalence estimates were logit-transformed and pooled using a random-effects model with restricted maximum likelihood estimation. Heterogeneity was quantified using I^2^ and Cochran’s Q. Subgroup and sensitivity analyses were performed by sex, methodological quality, diagnostic criteria, and geographic region. Publication bias was assessed using Egger’s test and the trim-and-fill procedure.

**Results:**

Fourteen cross-sectional studies involving 8,107 schoolteachers from 10 countries were included. The pooled prevalence of MetS was 30% (95% CI: 22–38%), with very high between-study heterogeneity (*I*^2^ =98.34%). Central obesity, dyslipidemia, and hypertension were the most frequently reported components. The highest pooled prevalence was observed in studies conducted in Asia (35, 95% CI: 25–45%), whereas the single European study reported the lowest prevalence (9, 95% CI: 8–10%).

**Conclusion:**

Across the included studies, approximately three in 10 schoolteachers met the diagnostic criteria for MetS, although prevalence estimates varied widely across settings. These findings support the integration of cardiometabolic risk assessment into occupational health services for schoolteachers and the development of workplace health promotion strategies focused on physical activity, healthy eating, and routine monitoring of metabolic health. Educational institutions and public health authorities should consider context-specific preventive approaches, while future longitudinal studies using standardized diagnostic criteria and detailed occupational exposure assessments are needed to strengthen the evidence base.

**Systematic review registration:**

https://www.crd.york.ac.uk/PROSPERO/view/CRD420251034493, CRD420251034493.

## 1 Introduction

Metabolic syndrome (MetS) is a major global public health concern characterized by the clustering of central obesity, hypertension, dyslipidemia, and impaired glucose metabolism (1, 2). Its prevalence varies across diagnostic criteria and geographic contexts, with recent global estimates ranging from 12.5 to 31.4% and reaching approximately 28.4% in 2023 (3, 4). MetS substantially increases the risk of cardiovascular disease, type 2 diabetes mellitus, and all-cause mortality, underscoring the importance of its prevention, early detection, and management (5–7).

Primary and secondary school teachers constitute a potentially vulnerable occupational group because their work is frequently characterized by high psychosocial demands, administrative burden, prolonged working hours, sedentary behavior, and limited opportunities for regular physical activity (8–13). Beyond its consequences for individual health, cardiometabolic disease among teachers may contribute to sickness absence, reduced work ability, presenteeism, and disruptions in workforce continuity, with potential implications for institutional functioning and educational quality (14, 15). Evidence from different regions has documented a high prevalence of hypertension, central obesity, physical inactivity, work-related stress, and other cardiovascular risk factors among teachers. In Latin America, the UNESCO Survey of Teachers’ Working Conditions and Health reported a prevalence of diagnosed hypertension of up to 39% in Chile, together with high levels of work-related stress and insufficient physical activity (13). Similarly, studies conducted in Europe, Asia, Africa, and South America have reported substantial levels of cardiovascular risk factors and metabolic abnormalities among teachers (16–20).

Several studies have reported a high prevalence of MetS risk factors among teachers, compared to other occupational groups (20–25). However, the available evidence is methodologically heterogeneous, with variable diagnostic criteria, dissimilar sampling strategies, and inconsistent assessment of occupational exposures, which limits comparisons between studies. The incorporation of occupational variables, such as workload, teaching hours, and associated psychosocial factors, is rarely reported.

Although several studies have reported the prevalence of MetS and its components among schoolteachers, the available evidence remains fragmented (18, 20–24). Existing studies provide individual prevalence estimates based on different diagnostic criteria, sampling strategies, and geographic contexts, thereby limiting direct comparisons across populations. Moreover, no pooled estimate of MetS prevalence among primary and secondary schoolteachers is currently available, and potential sources of variability, including sex, geographic region, methodological quality, and diagnostic criteria, have not been systematically examined. Occupational determinants such as teaching workload, psychosocial stress, sedentary behavior, physical activity, and working conditions have also been inconsistently assessed (17, 20, 23, 26), preventing a comprehensive understanding of their potential contribution to cardiometabolic risk in this occupational group.

To our knowledge, this is the first systematic review and meta-analysis to synthesize and quantify the prevalence of MetS specifically among primary and secondary schoolteachers. In addition to providing an overall pooled estimate, this review examines variation by sex, geographic region, diagnostic criteria, and methodological quality, and summarizes the occupational and lifestyle-related factors reported in the available literature. By integrating evidence from diverse geographic settings, this study provides a more comprehensive assessment of the cardiometabolic burden among schoolteachers.

Therefore, the primary objective of this systematic review and meta-analysis was to estimate the pooled prevalence of MetS among primary and secondary schoolteachers. The secondary objectives were to evaluate variations in prevalence by sex, geographic region, diagnostic criteria, and methodological quality, and to summarize the occupational and lifestyle-related factors reported to be associated with MetS in the included studies.

## 2 Methods

### 2.1 Protocol and registration

This systematic review and meta-analysis followed the PRISMA 2020 guidelines (27) and was prospectively registered in PROSPERO (CRD420251034493). The search strategy was designed following the PECOS framework (Participants, Exposure, Comparator, Outcomes, Study Design) (see Table 1).

**Table 1:** PECOS criteria for study inclusion and exclusion.

Component	Criteria
Participants	Primary and secondary school teachers aged 18–70 years
Exposure	Active practice as a primary or secondary school teacher
Comparator	Not applicable
Outcomes	MetS was assessed using a validated diagnostic definition, including the IDF, NCEP ATP III, AHA/NHLBI, WHO, or Joint Interim Statement harmonized criteria.
Study design	Cohort studies, cross-sectional studies, case–control studies, randomized and non-randomized controlled trials

ATP, Adult Treatment Panel; AHA, American Heart Association; IDF, International Diabetes Federation; NCEP, National Cholesterol Education Program; NHLBI, National Heart, Lung, and Blood Institute; WHO, World Health Organization.

### 2.2 Inclusion and exclusion criteria

Inclusion criteria: original research articles and studies conducted in humans with participants aged 18 to 70 years that report the prevalence of MetS using a validated diagnostic criterion (see Table 2).

**Table 2:** Diagnostic criteria for metabolic syndrome.

Risk factor	JIS/IDF–AHA/NHLBI (2009)	IDF (2005)	AHA/NHLBI (2004)	NCEP ATP-III (2001)	WHO (1998)*
Diagnostic criterion	≥3 of 5 components	Central obesity + ≥ 2 components	≥3 of 5 components	≥3 of 5 components	Insulin resistance + ≥ 2 components
BMI	N/A	N/A	N/A	N/A	≥30 kg/m^2^
Central obesity (WC)	Ethnic-specific cut-offs (IDF values)	Europeans: ≥94/≥80 cm; Asians: ≥90/≥80 cm	≥102 cm (M); ≥88 cm (F)	≥102 cm (M); ≥88 cm (F)	WHR ≥ 0.90 (M); ≥0.85 (F)
Fasting glucose	≥100 mg/dL or T2DM Rx	≥100 mg/dL or T2DM Rx	≥100 mg/dL or T2DM Rx	≥110 mg/dL	≥100 mg/dL (IR criterion)
Triglycerides	≥150 mg/dL or Rx	≥150 mg/dL or Rx	≥150 mg/dL or Rx	≥150 mg/dL	≥150 mg/dL
HDL-cholesterol	<40 mg/dL (M); <50 mg/dL (F) or Rx	<40 mg/dL (M); <50 mg/dL (F) or Rx	<40 mg/dL (M); <50 mg/dL (F) or Rx	<40 mg/dL (M); <50 mg/dL (F)	<40 mg/dL (M); <50 mg/dL (F)
Blood pressure	≥130/85 mmHg or Rx	≥130/85 mmHg or Rx	≥130/85 mmHg or Rx	≥130/85 mmHg	≥140/90 mmHg

JIS, Joint Interim Statement; IDF, International Diabetes Federation; AHA, American Heart Association; NHLBI, National Heart, Lung, and Blood Institute; NCEP, National Cholesterol Education Program; ATP, Adult Treatment Panel; WHO, World Health Organization; BMI, Body Mass Index; WC, waist circumference; WHR, waist-to-hip ratio; M, male; F, female; T2DM, type 2 diabetes mellitus; IR, insulin resistance; Rx, pharmacological treatment. *The WHO criteria were not used in any study included in this meta-analysis.

Exclusion criteria: reviews, meta-analyses, letters to the editor, conference abstracts, case reports, studies involving other categories of educators (e.g., university professors, instructors at technical or vocational institutes), and studies focused on unrelated pathologies, and grey literature, including theses, dissertations, conference proceedings, government reports, institutional documents, and preprints.

### 2.3 Search strategy

A systematic literature search was conducted in PubMed, Scopus, and Web of Science from their inception until April 27, 2026. Terms related to “metabolic syndrome” and “teachers” were combined using Boolean operators (Table 3). No restrictions were applied by year of publication or language.

**Table 3:** Search strategy used in the review (total records = 455).

Database	Search terms	Records
PubMed	(“metabolic syndrome”[MeSH Terms] OR (“metabolic”[All Fields] AND “syndrome”[All Fields])) AND (“schoolteachers”[MeSH Terms] OR (“school”[All Fields] AND “teachers”[All Fields]) OR “school teachers”[All Fields])	*n* = 61
Scopus	(“metabolic syndrome” OR (metabolic AND syndrome)) AND (“schoolteachers” OR (school AND teachers))	*n* = 110
Web of Science	(“metabolic syndrome” OR (metabolic AND syndrome) OR “syndrome X”) AND (“schoolteachers” OR (school AND teachers) OR educators OR “teaching staff”)	*n* = 284

### 2.4 Study selection

**Figure 1:**
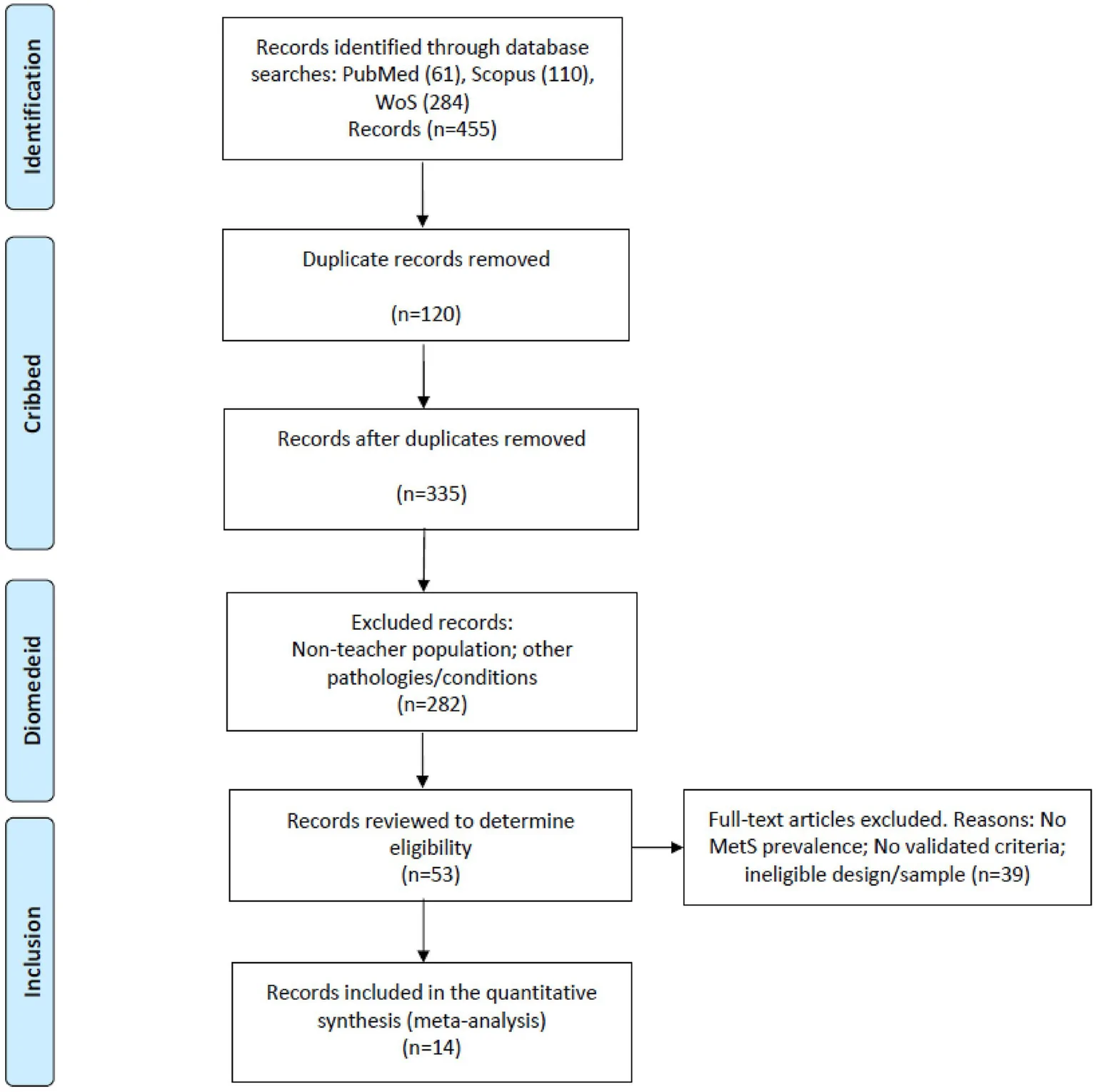
PRISMA 2020 flow diagram showing the study selection process. Records identified through database searching (PubMed, *n* = 61; Scopus, *n* = 110; Web of Science, *n* = 284), with final inclusion of 14 cross-sectional studies in the systematic review and meta-analysis.

Two reviewers independently selected titles, abstracts, and full texts (TY and PH). Discrepancies were resolved by consensus with a third reviewer (PLA). The selection process is detailed in the PRISMA 2020 flow diagram (Figure 1).

### 2.5 Data extraction

A standardized data extraction form was used to collect information from each study, including country, sample size, participant characteristics (age and sex), study design, diagnostic criteria used, MetS prevalence, component-level data, and associated variables. For studies reporting mixed occupational populations (e.g., (21)), only teacher data were extracted.

### 2.6 Methodological quality assessment

Methodological quality was assessed using the NHLBI Quality Assessment Tool for Observational Cohort and Cross-Sectional Studies, which evaluates 14 items. The NHLBI tool was selected because it evaluates methodological domains relevant to observational prevalence studies, including participant selection, sample definition, exposure and outcome measurement, control of confounding, and completeness of reporting. Its use also allowed the application of a consistent quality assessment framework across studies with heterogeneous sampling strategies and reporting practices. Each criterion was rated as “Yes,” “No,” or “Other” (CD = Not determinable; NA = Not applicable; NR = Not reported). Studies were classified as good quality (≥8/14) or moderate-to-low quality (<8/14). Two independent reviewers assessed methodological quality, and discrepancies were resolved by consensus.

### 2.7 Statistical analysis

A random-effects meta-analysis was performed using the REML estimator in Stata 16. Crude prevalence proportions were logit-transformed to stabilize variance. A pooled meta-analysis was considered appropriate despite anticipated between-study heterogeneity because the included studies addressed the same underlying outcome, specifically the prevalence of MetS among primary and secondary schoolteachers. A random-effects model was used to estimate the average prevalence across heterogeneous settings rather than a single uniform prevalence applicable to all populations. Accordingly, the pooled estimate was interpreted alongside measures of heterogeneity, subgroup analyses, meta-regression, and leave-one-out sensitivity analyses. Subgroup and sensitivity analyses were conducted to explore clinically and methodologically plausible sources of heterogeneity, including sex, methodological quality, diagnostic criteria, and geographic region. For studies reporting more than one MetS definition, only one estimate was included in the primary synthesis to avoid double counting, prioritizing the criterion identified as primary by the original authors or the definition most consistently described in the Methods section. Heterogeneity was quantified using Cochran’s Q test and the I^2^ statistic. An I^2^ > 75% was considered indicative of substantial heterogeneity (28). Forest plots were generated for the overall analysis and for all subgroup analyses.

Publication bias was assessed using Egger’s linear regression test and the trim-and-fill procedure. The results of Egger’s test were interpreted cautiously because the statistical power of tests for funnel plot asymmetry may be limited when the number of available studies is small. Sensitivity analyses were performed for: (1) methodological quality (for meta-regression, dichotomized as “good (≥8) vs. moderate/low (<8)” according to NHLBI); (2) diagnostic criteria (NCEP ATP-III vs. IDF vs. JIS); and (3) geographic region (Asia, Africa, South America, and Europe). Meta-regression models were estimated using REML random effects with these moderators. Statistical significance was set at *p* < 0.05.

## 3 Results

### 3.1 Study selection

The database search identified 455 articles (PubMed: 61; Scopus: 110; Web of Science: 284). After removing duplicate studies, 14 cross-sectional studies with 8,107 teachers met all the inclusion criteria and were included in the meta-analysis. Figure 1 shows the step-by-step study selection process.

### 3.2 Characteristics of the included studies

The 14 included studies were conducted between 2016 and 2025 in 10 countries: India (2), South Africa (2), Nigeria (2), Indonesia (2), Malaysia (1), Iran (1), Saudi Arabia (1), Spain (1), Egypt (1), and Brazil (1). Sample sizes ranged from 43 (29) to 4,738 (30). Participants were predominantly female (weighted mean ~68%), and the mean age was over 40 years in all studies that reported these data (k = 9; range: 41.0–51.3 years). All studies were cross-sectional in design (see Table 4).

**Table 4:** Characteristics of the included studies.

Reference (Country)	*n*	Sex	Average age (±SD)	Diagnostic criteria	BMI/nutritional status	MetS Prevalence (%)	Quality (NHLBI)
Bajaber et al. (32) (Saudi Arabia)	300	F	NR (range 30–55)	NCEP ATP-III (AHA-NHLBI)	Obese 17% (51/300)	28.0	9/14—Good
Narayanappa (23) (India)	300	M/F	44.7 ± 9.5	IDF	Mean: 25.7 ± 4.9 kg/m^2^ Mean with MetS: 28.4 ± 4.1 kg/m^2^ / Both overweight	38.3	7/14—Moderate-low
Akintunde and Oloyede (21) (Nigeria)*	83	M/F	45.7 ± 8.2	JIS Harmonized, (also IDF/WHO)	27.2 ± 5.1 kg/m^2^ / overweight	7.2 (IDF 6.0; WHO 1.2)	5/14—Moderate-low
Lee et al. (31) (Malaysia)	1,168	M/F	42.51 ± 0.49	JIS Harmonized	NR / Non-obese adults (normal weight + overweight only)	17.7	5/14—Moderate-low
Rachmah and Utari (25) (Indonesia)	138	M/F	45.0 ± 10.07	NCEP ATP-III	NR / NR	24.6	9/14 — Good
Oliveira et al. (24) (Brazil)	200	M/F	43.0 ± 10.2	IDF	58% overweight	20.0	8/14 — Good
Ayogu et al. (29) (Nigeria)**	43	M/F	NR	IDF	NR / NR	23.3	10/14—Good
Hasan et al. (33) (Indonesia)	129	M/F	49.8 ± 7.4	IDF***	Obese sample: 100%	39.5	9/14—Good
López González et al. (30) (Spain)	4,738	M/F	41.0 ± 10.4	NCEP ATP-III (also IDF and JIS)	NR/13.0% obesity	9.2**** (IDF 7.9; JIS 13.2)	10/14—Good
Veldsman et al. (20) (South Africa)	216	M/F	51.25 ± 8.11†	JIS Harmonized	30.3 ± 6.8 kg/m^2^/Obesity	29.2	8/14—Good
Monica et al. (22) (India)	256	F	NR	JIS Harmonized	NR / 68.4% (overweight + obesity).	39.5	7/14 — Moderate-low
Jowshan et al. (34) (Iran)	97	F	43.87 ± 7.14	NCEP ATP-III	27.06 ± 4.41/overweight	59.8	10/14—Good
Joubert et al. (17) (South Africa)	158	M/F	47.2 ± 10.7	JIS Harmonized	NR / NR	58.2	11/14—Good
Ramadan et al. (18) (Egypt)	281	M/F	47.26 ± 5.75	IDF	Obesity, defined as a BMI of ≥30 kg/m^2^, was present in 34.6% of the participants	25.8	8/14—Good

*Akintunde and Oloyede (21) reports MetS prevalence according to WHO, IDF, and Joint Interim Statement (JIS) criteria; for this meta-analysis, the harmonized definition (JIS) was used, and only the group of teachers (*n* = 83) was included. ** Ayogu et al. (29) included teachers and bank employees; the prevalence of metabolic syndrome (IDF 2006) was estimated in a subsample with lipid profiles (*n* = 50). For this meta-analysis, only the teachers from this subsample were used (*n* = 43; 10 cases, 23.3%). *** Hasan et al. (33) stated in the abstract that the Joint Interim Statement criteria were used; however, the Methods section described the definition of metabolic syndrome as based on the International Diabetes Federation criteria, using Asian-specific waist circumference cut-offs and a diagnosis based on the presence of at least three components. In accordance with the predefined harmonization rule, the study was classified under the IDF criterion. BMI/nutritional status was reported as presented in the original studies because of substantial heterogeneity in measurement and reporting methods. Values may correspond to mean BMI, prevalence of overweight or obesity, or the nutritional profile of the selected sample. NR: not reported. M, Male; F, Female; NR, Not reported; IDF, International Diabetes Federation; NCEP, National Cholesterol Education Program; ATP, Adult Treatment Panel; JIS, Joint Interim Statement; BMI, Body Mass Index. ^†^Mean age reported for teachers with metabolic syndrome, rather than for the total study sample.

### 3.3 Prevalence of MetS—main meta-analysis

**Figure 2:**
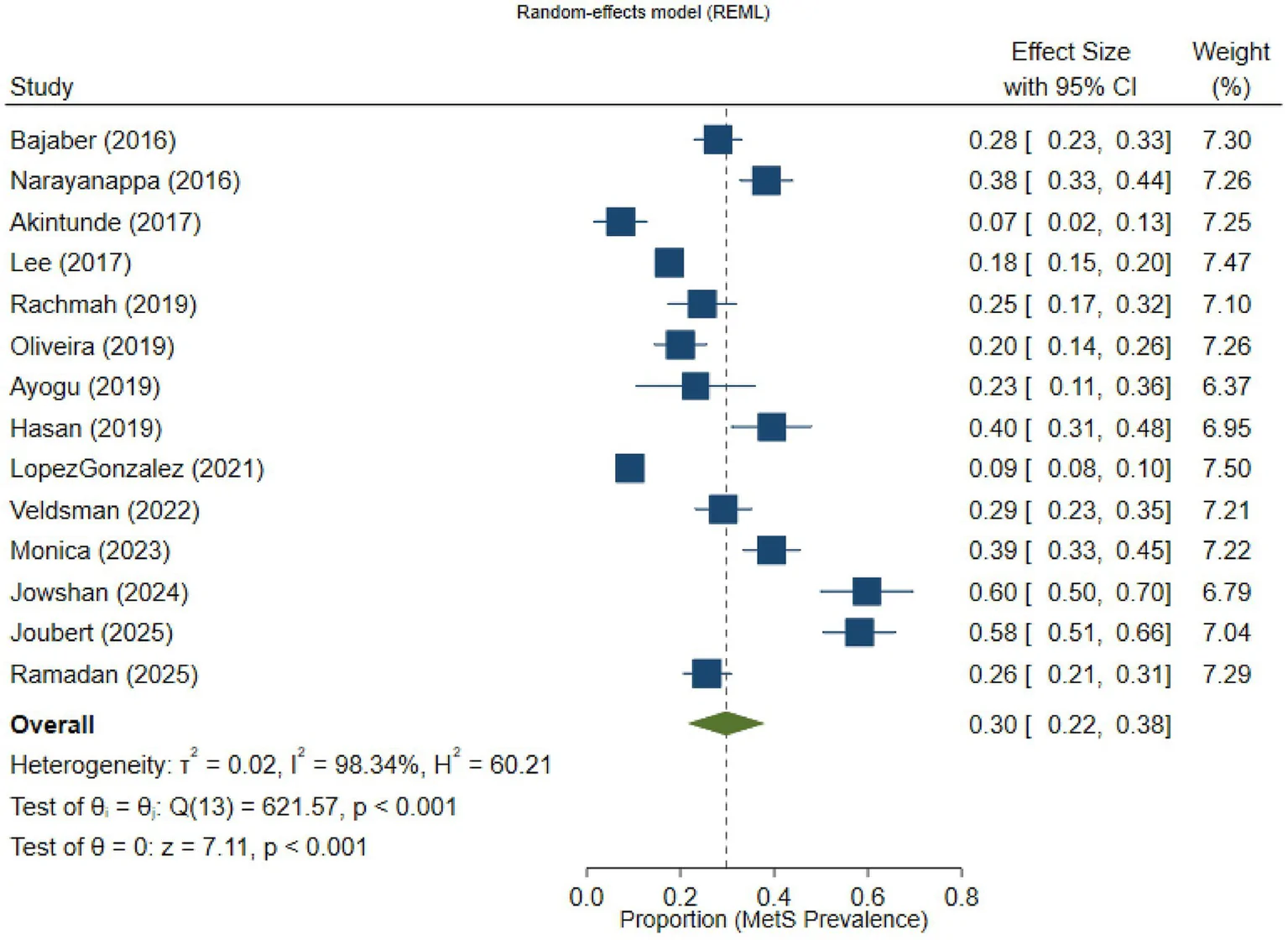
Forest plot of the pooled prevalence of metabolic syndrome among schoolteachers worldwide (*k* = 14; *n* = 8,107). Each square represents the point estimate of individual studies, with the size proportional to the study weight; horizontal lines represent 95% confidence intervals; the diamond represents the pooled random-effects estimate. The dashed vertical line indicates the pooled effect size. *ES*: Effect size (proportion); *CI*: Confidence interval; *I*^2^: Inconsistency index. Random-effects model (REML). Pooled *ES* = 0.30 (95% *CI*: 0.22–0.38); *I*^2^ = 98.34%, *p* < 0.001.

The pooled prevalence of MetS across the 14 studies was 30% (95% CI: 22–38%) using a random-effects model (Figure 2). High heterogeneity was observed (*I*^2^ = 98.34%; Q = 621.57; df = 13; *p* < 0.001), indicating significant variability between studies. Individual prevalences ranged from 7.2 to 59.8%.

### 3.4 Prevalence of MetS—subgroup analysis by sex

The subgroup analysis by sex included 9 studies that reported sex-disaggregated data (see Table 5). The primary analysis was restricted to the five studies that used the IDF criterion (k = 5; men: *n* = 312; women: *n* = 649), while the sensitivity analysis included all nine studies regardless of the diagnostic criterion used—IDF, JIS, and NCEP ATP-III— (k = 9; men: *n* = 2,457; women: *n* = 4,784). The study by López González et al. (30) was classified according to the NCEP ATP-III criterion, which the authors declared as the main criterion, and was included exclusively in the sensitivity analysis. The studies by Lee et al. (31), Veldsman et al. (20), and Joubert et al. (17) were classified under the JIS (Harmonization) criterion and were also included only in the sensitivity analysis.

**Table 5:** Subgroup analysis by sex: pooled prevalence of metabolic syndrome among male and female schoolteachers.

Subgroup	*k*	*N*	ES pooled	95% CI	*I* ^2^	Q (df)	*p*-het	τ^2^
Primary—Male (IDF)	5	312	0.276	0.111–0.442	92.23%	43.43 (4)	<0.001	0.032
Primary—Female (IDF)	5	649	0.305	0.225–0.385	78.97%	21.25 (4)	<0.001	0.006
Sensitivity—Male (IDF + JIS + NCEP)	9	2,457	0.323	0.186–0.460	97.27%	159.09 (8)	<0.001	0.041
Sensitivity—Female (IDF + JIS + NCEP)	9	4,784	0.271	0.169–0.373	98.21%	317.84 (8)	<0.001	0.023

All estimates are based on random-effects models (REML). Between subgroups in the primary analysis (IDF criterion), Wald *χ*^2^ = 0.18, df = 1, *p* = 0.670 was observed. Between subgroups in the sensitivity analysis (combined IDF+JIS+NCEP criteria), Wald *χ*^2^ = 0.31 (df = 1), *p* = 0.580, indicating no significant differences between sexes.

In the primary analysis (k = 5, IDF criterion), the pooled prevalence was 27.6% (95% CI: 11.1–44.2%) in male teachers and 30.5% (95% CI: 22.5–38.5%) in female teachers. Both subgroups showed statistically significant and substantial heterogeneity (men: *I*^2^ = 92.23%, Q = 43.43, df = 4, *p* < 0.001, τ^2^ = 0.032; women: *I*^2^ = 78.97%, Q = 21.25, df = 4, *p* < 0.001, τ^2^ = 0.006). The test for heterogeneity between subgroups was not statistically significant (χ^2^ Wald = 0.18, df = 1, *p* = 0.670), indicating no statistically significant differences in the prevalence of MetS between male and female teachers according to the IDF criterion.

In the sensitivity analysis (k = 9, combined IDF, JIS, and NCEP criteria), pooled prevalences were 32.3% (95% CI: 18.6–46.0%) in men and 27.1% (95% CI: 16.9–37.3%) in women (men: *I*^2^ = 97.27%, Q = 159.09, df = 8, *p* < 0.001, τ^2^ = 0.041; women: *I*^2^ = 98.21%, Q = 317.84, df = 8, *p* < 0.001, τ^2^ = 0.023). Heterogeneity between subgroups remained non-significant (χ^2^ Wald = 0.31, df = 1, *p* = 0.580), confirming the absence of systematic differences by sex, regardless of the diagnostic criteria used. The inclusion of studies using JIS and NCEP criteria increased the pooled prevalence in the male subgroup by approximately 4.7 percentage points compared to the primary analysis, reflecting the notably high prevalences reported by Veldsman et al. (20) and Joubert et al. (17) in male teachers in South Africa (46.2 and 72.0%, respectively). The study by Ayogu et al. (29) reported 0 events in the male subgroup (*n* = 23), a continuity correction (cases = 1, total = 24) was required for its inclusion in the model.

### 3.5 Components of MetS

Central obesity was the most prevalent component of MetS in virtually all included studies, with frequencies ranging from 50 to 99% of participants depending on the study population (22, 24), although not all studies reported data for all MetS components. Dyslipidemia was generally the second most prevalent component, followed by hypertension and impaired fasting glucose, which had lower prevalences.

### 3.6 Reported factors associated with MetS

Among the studies that reported associated factors, physical inactivity emerged as the most consistent behavioral risk factor for MetS (17, 20, 25, 26). Joubert et al. (17) reported that each additional hour per week of moderate-to-vigorous physical activity was associated with a 27% lower likelihood of MetS (OR = 0.73; 95% CI: 0.55–0.95). Age was a significant positive predictor in two studies (17, 23). High total fat intake (>35% of energy) was associated with higher odds of MetS (OR = 3.37; 95% CI: 1.03–11.96), whereas higher socioeconomic status was associated with lower odds of MetS in the South African context (OR = 0.14; 95% CI: 0.02–0.72) (17). Work-related stress was formally assessed in only one study included in the meta-analysis (23), which reported a high prevalence of moderate-to-severe stress among teachers.

### 3.7 Methodological quality assessment

Table 6 presents the methodological quality scores assessed using the NHLBI tool in the 14 included studies. Ten studies scored ≥8/14 and were classified as good quality. The remaining four studies scored <8/14 and were classified as moderate-to-low quality for the purposes of subgroup analysis and meta-regression. No study scored below 5/14. The most frequent methodological limitations included: (1) lack of a justified sample size calculation, (2) convenience or purposive sampling strategies, (3) lack of adjustment for confounding variables, and (4) limited reporting of participation rates and loss to follow-up. Although methodological quality did not significantly moderate the pooled prevalence, the predominance of limitations related to confounding control, sampling, and reporting was considered when interpreting the certainty and generalizability of the findings.

**Table 6:** Methodological quality assessment (NHLBI).

Study	Type	NHLBI score (14)	Quality	Sample strategy
Bajaber et al. (32)	Cross-sectional	9/14	Good	Random sampling
Narayanappa (23)	Cross-sectional	7/14	Moderate-low	Multistage cluster sampling
Akintunde and Oloyede (21)	Cross-sectional	5/14	Moderate-low	Stratified random sampling
Lee et al. (31)	Cross-sectional	5/14	Moderate-low	Multistage cluster sampling
Rachmah and Utari (25)	Cross-sectional	9/14	Good	Multistage cluster sampling
Oliveira et al. (24)	Cross-sectional	8/14	Good	Random sampling
Ayogu et al. (29)	Cross-sectional	10/14	Good	Multistage cluster sampling
Hasan et al. (33)	Cross-sectional	9/14	Good	Non-probability sampling
López González et al. (30)	Cross-sectional	10/14	Good	Multicenter registry
Veldsman et al. (20)	Cross-sectional	8/14	Good	Consecutive sampling
Monica et al. (22)	Cross-sectional	7/14	Moderate-low	Multistage cluster sampling
Jowshan et al. (34)	Cross-sectional	10/14	Good	Multistage cluster sampling
Joubert et al. (17)	Cross-sectional	11/14	Good	Consecutive sampling
Ramadan et al. (18)	Cross-sectional	8/14	Good	Simple random sampling

NHLBI: National Heart, Lung, and Blood Institute. Classification criteria: Good ≥8/14; Moderate-low <8/14.

### 3.8 Publication bias

**Figure 3:**
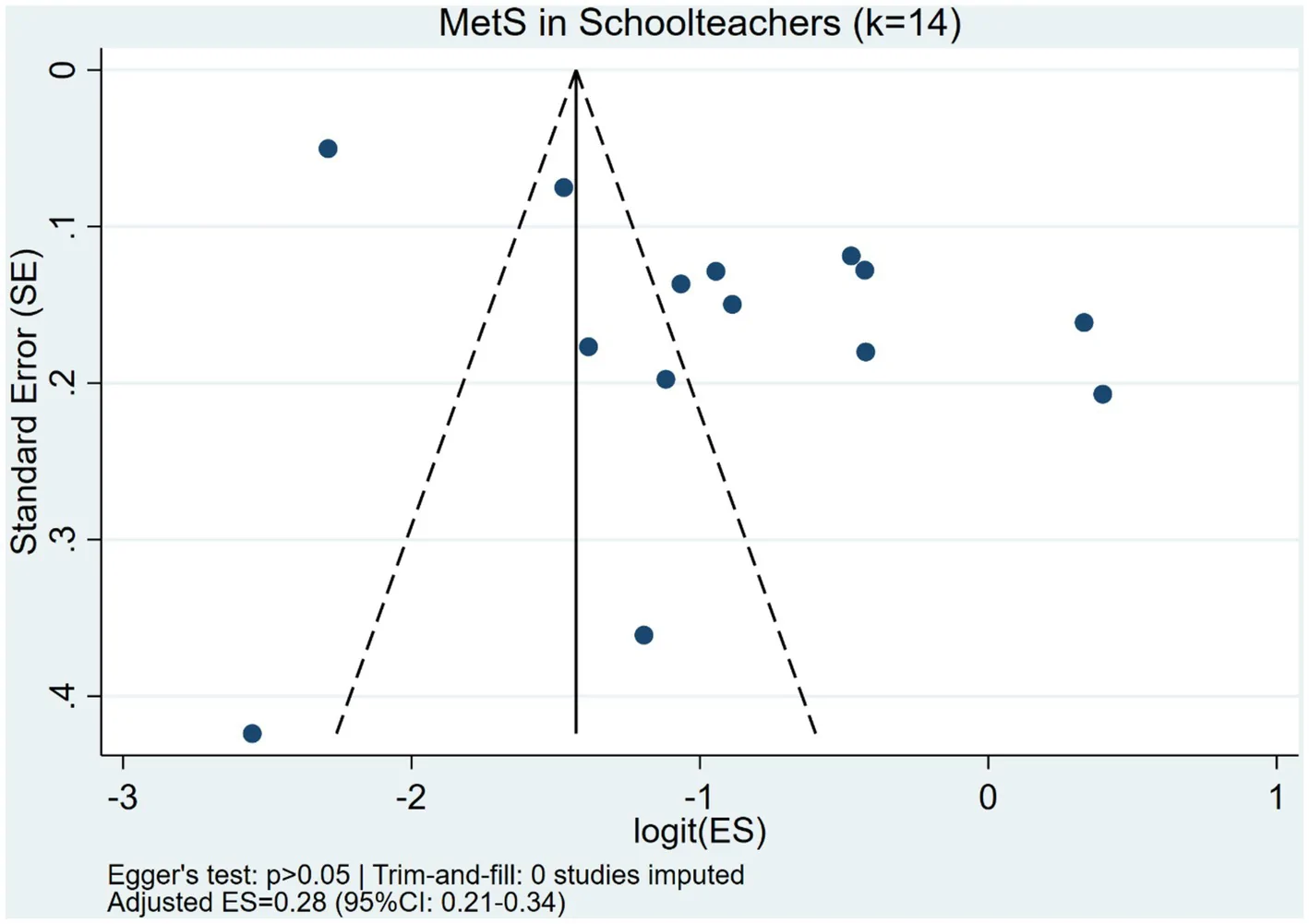
Funnel plot with pseudo 95% confidence limits for assessment of publication bias (*k* = 14). The *x*-axis represents the logit-transformed effect size (logit *ES*); the *y*-axis represents the standard error (*SE*) of each study estimate, with smaller *SE* (higher precision) at the top. The vertical solid line indicates the pooled effect estimate; the dashed lines represent the pseudo-95% confidence interval. Egger’s test revealed no statistically significant asymmetry (*p* > 0.05), and the trim-and-fill procedure imputed 0 additional studies, with the adjusted pooled estimate remaining stable at *ES* = 0.28 (95% *CI*: 0.21–0.34). The slight rightward asymmetry may partly reflect between-study heterogeneity, including geographic variation, rather than publication bias alone.

Publication bias was assessed using Egger’s linear regression test and the trim-and-fill procedure on the 14 included studies (Figure 3). Egger’s test did not detect statistically significant skewness in the funnel plot (*p* > 0.05), suggesting no systematic publication bias. However, this result should be interpreted cautiously because only 14 studies were available, limiting the statistical power of the test and preventing publication bias from being conclusively excluded. The trim-and-fill procedure did not impute additional studies, and the adjusted pooled estimate remained stable at 28% (95% CI: 21–34%). The slight rightward skewness observed in the funnel plot may partly reflect the substantial between-study heterogeneity, including geographic variation.

### 3.9 Sensitivity analysis

#### 3.9.1 By methodological quality

**Figure 4:**
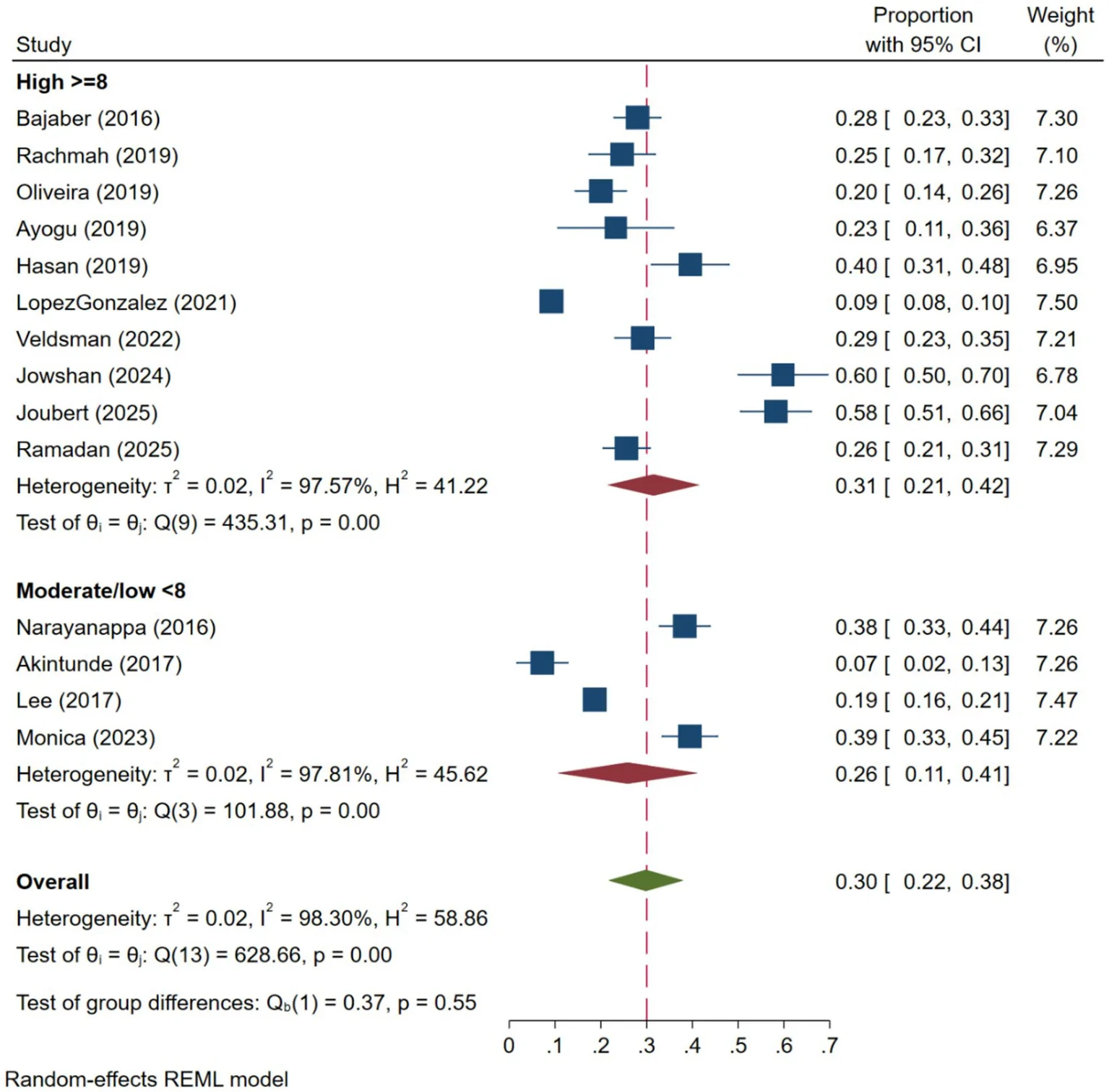
Forest plot of subgroup analysis by methodological quality according to the NHLBI tool. Studies classified as good quality (NHLBI ≥ 8/14; *k* = 10) showed a pooled prevalence of 31% (95% CI: 21–42%; *I*^2^ = 97.57%; *τ*^2^ = 0.02), whereas studies classified as moderate-to-low quality (NHLBI <8/14; *k* = 4) showed a pooled prevalence of 26% (95% CI: 11–41%; *I*^2^ = 97.81%; *τ*^2^ = 0.02). The difference between subgroups was not statistically significant (*Q*_b_ = 0.37; *df* = 1; *p* = 0.55). ES, effect size; CI, confidence interval; *I*^2^, inconsistency index; *τ*^2^, between-study variance. Random-effects REML model.

Figure 4 presents the subgroup analysis by methodological quality. Ten studies were classified as good quality (NHLBI ≥8/14) (17, 18, 20, 24, 25, 29, 30, 32–34) and reported a pooled prevalence of 31% (95% CI: 21–42%; *I*^2^ = 97.57%; τ^2^ = 0.02). Four studies were classified as moderate-to-low quality (NHLBI <8/14) (21–23, 31) and reported a pooled prevalence of 26% (95% CI: 11–41%; *I*^2^ = 97.81%; τ^2^ = 0.02). The difference between subgroups was not statistically significant (Qb = 0.37; df = 1; *p* = 0.55), indicating that methodological quality did not significantly moderate the prevalence of MetS.

#### 3.9.2 By diagnostic criterion

**Figure 5:**
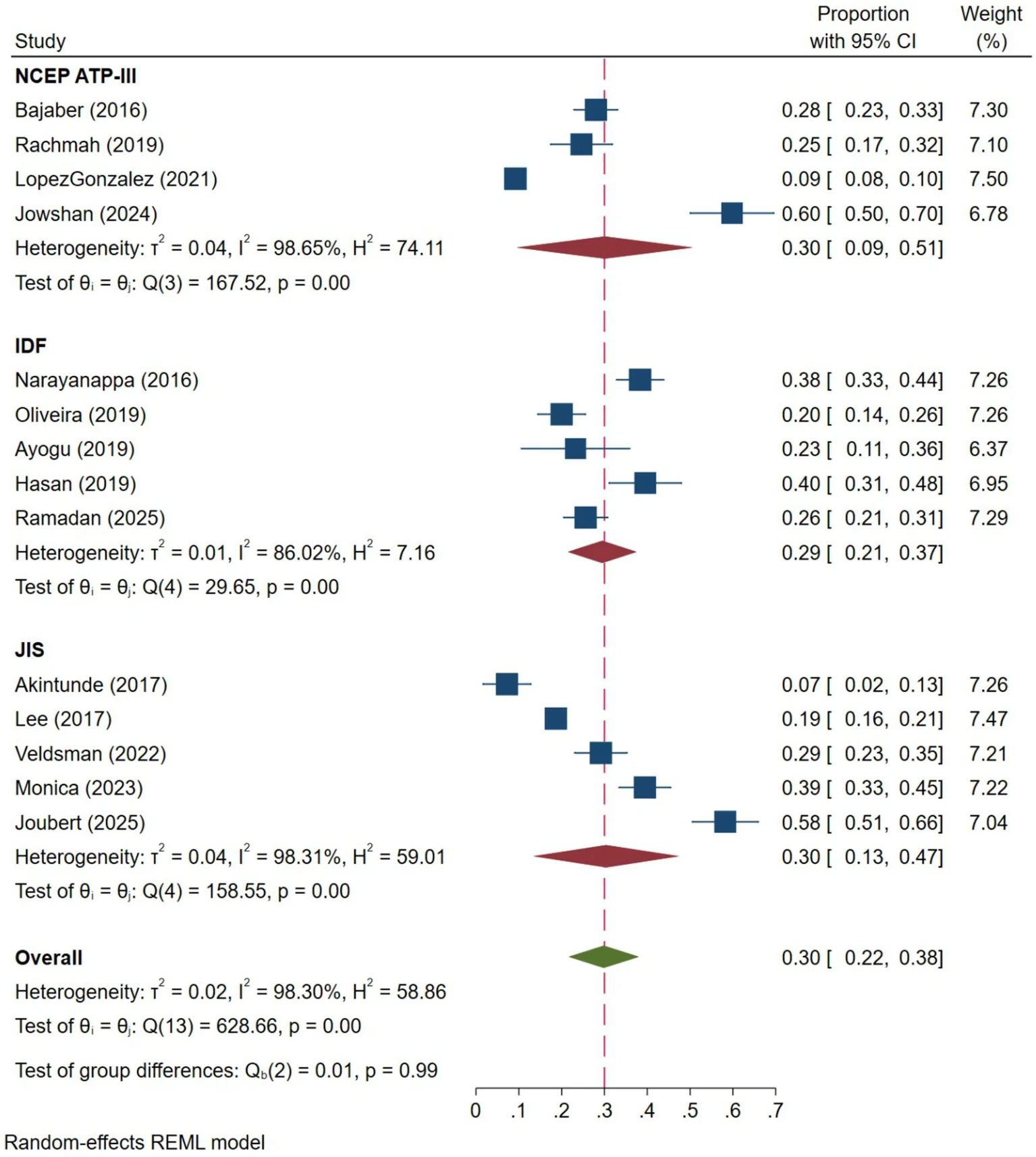
Forest plot of subgroup analysis by diagnostic criterion. Subgroups: NCEP ATP-III (*k* = 4), IDF (*k* = 5), and JIS (*k* = 5). Pooled prevalences: NCEP ATP-III, *ES* = 0.30 (95% CI: 0.10–0.51; *I*^2^ = 98.7%; τ^2^ = 0.04); IDF, *ES* = 0.29 (95% CI: 0.22–0.38; *I*^2^ = 86.0%; τ^2^ = 0.01); JIS, *ES* = 0.30 (95% CI: 0.13–0.47; *I*^2^ = 98.3%; τ^2^ = 0.04). Between-subgroup heterogeneity: Q_b_ = 0.01, *df* = 2, *p* = 0.99. *ES*, effect size (proportion); *CI*, confidence interval; I^2^, inconsistency index; τ^2^, between-study variance. Random-effects REML model.

In the stratification by diagnostic criterion, the pooled prevalences were very similar across criteria (Figure 5). Studies that used the NCEP ATP-III criterion (k = 4) showed a pooled prevalence of MetS of 30% (95% CI: 9.5–50.6%; *I*^2^ = 98.7%; τ^2^ = 0.04), while those that applied the IDF criterion (k = 5) reported 29% (95% CI: 21.5–37.5%; *I*^2^ = 86.0%; τ^2^ = 0.01) and those that used the JIS criterion (k = 5) reported 30% (95% CI: 13.3–47.4%; *I*^2^ = 98.3%; τ^2^ = 0.04). The test of heterogeneity between subgroups was not statistically significant (Qb = 0.01; df = 2; *p* = 0.995), indicating that, under a REML random-effects model, the diagnostic criterion does not moderate the prevalence of MetS among teachers.

#### 3.9.3 By geographic region

**Figure 6:**
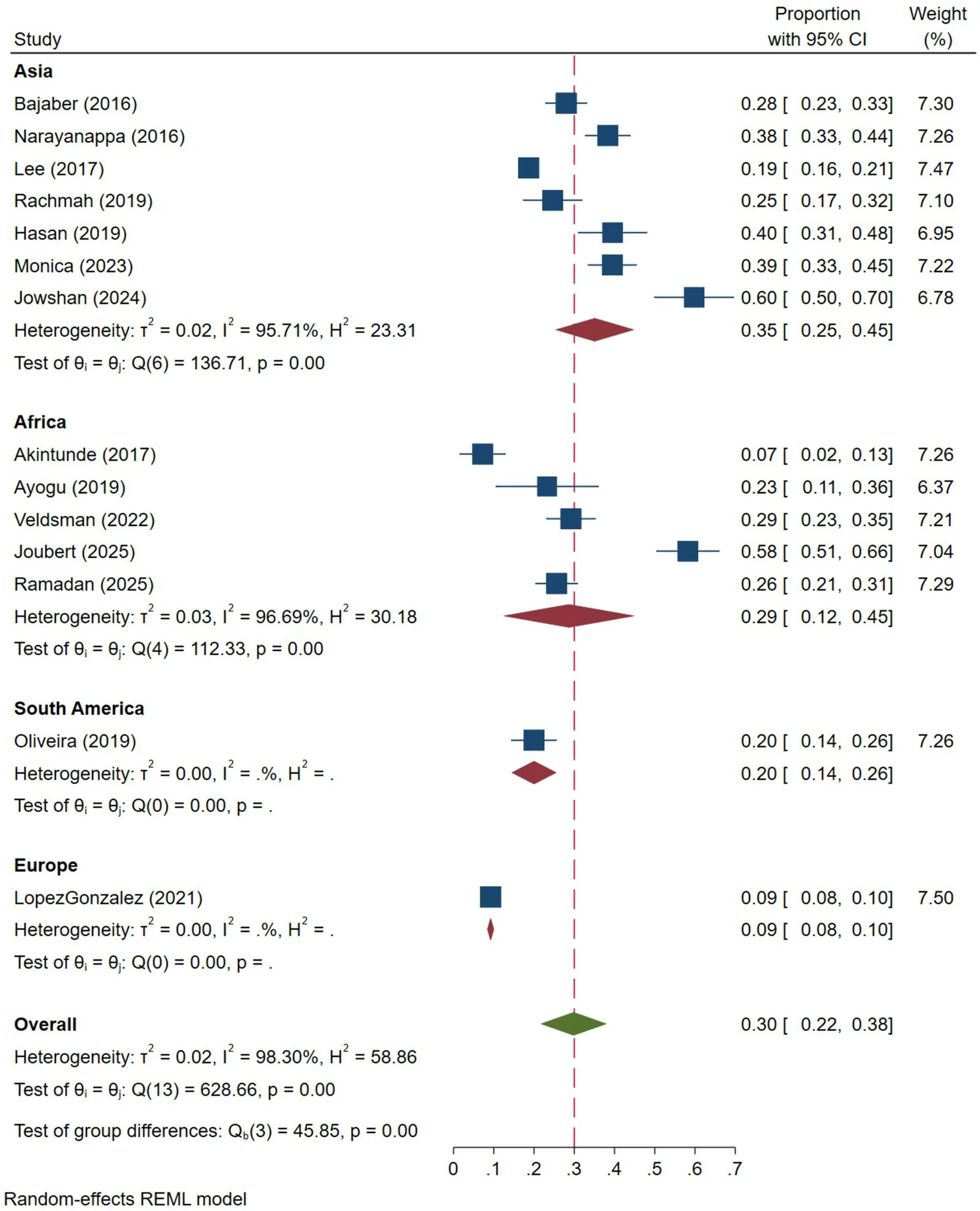
Forest plot of subgroup analysis by geographic region. Subgroups: Asia (*k* = 7), Africa (*k* = 5), South America (*k* = 1), and Europe (*k* = 1). Pooled prevalences: Asia, *ES* = 0.35 (95% *CI*: 0.25–0.45; *I*^2^ = 95.7%; τ^2^ = 0.02); Africa, *ES* = 0.29 (95% *CI*: 0.12–0.45; *I*^2^ = 96.7%; τ^2^ = 0.03); South America, *ES* = 0.20 (95% *CI*: 0.15–0.26); Europe, *ES* = 0.09 (95% *CI*: 0.08–0.10). Between-subgroup heterogeneity: Q_b_ = 45.85, *df* = 3, *p* < 0.001. *ES*, effect size (proportion); *CI*, confidence interval; I^2^, inconsistency index; τ^2^, between-study variance. Random-effects REML model.

In the sensitivity analysis by geographic region, clear differences in the prevalence of MetS among teachers were observed (Figure 6). Studies conducted in Asia (k = 7) showed a pooled prevalence of 35% (95% CI: 25.3–44.8%; *I*^2^ = 95.7%; τ^2^ = 0.02), while African studies (k = 5) reported 29% (95% CI: 12.3–45.0%; *I*^2^ = 96.7%; τ^2^ = 0.03). In South America (k = 1, Brazil) the prevalence was 20% (95% CI: 14.5–25.5%), and in Europe (k = 1, Spain) it was 9.2% (95% CI: 8.4–10.0%). Heterogeneity between subgroups was statistically significant (Qb = 45.85; df = 3; *p* < 0.001), indicating that geographic region was a relevant moderator of MetS prevalence in a REML random-effects model.

#### 3.9.4 Leave-one-out sensitivity analysis

The leave-one-out analysis confirmed the robustness of the overall pooled estimate (Table 7). Individual exclusion of each study resulted in prevalences ranging from 27.6 to 31.6%, with no substantial deviation from the overall estimate of 29.8% (95% CI: 21.7–38.0%) obtained using a REML random-effects model. The most significant changes were observed when excluding Jowshan et al. (34) or Joubert et al. (17), which reduced the pooled estimate to 27.6%, and when excluding Akintunde and Oloyede (21), which increased it to 31.6%. Excluding the large study by López González et al. (30) also slightly increased the prevalence (≈31.5%) and was associated with a relative reduction in heterogeneity, although the I^2^ remained high. In the other iterations, the variations were minimal, confirming that no single study is solely responsible for the observed variability or compromises the stability of the overall estimate.

**Table 7:** Leave-one-out sensitivity analysis of the pooled prevalence of metabolic syndrome in teachers.

Study excluded	*k*	N Remaining	ES pooled	95% CI	*I*^2^ (%) approx.	Δ vs. global
Monica et al. (22)	13	7,851	0.2910	0.2041–0.3780	~98	↓ Mild
Veldsman et al. (20)	13	7,891	0.2991	0.2107–0.3875	~98	→ Stable
Akintunde and Oloyede (21)	13	8,024	0.3158	0.2356–0.3960	~98	**↑Notable increase**
Narayanappa (23)	13	7,807	0.2919	0.2046–0.3792	~98	→ Stable
Lee et al. (31)	13	6,939	0.3075	0.2209–0.3941	~98	→ Stable
Rachmah and Utari (25)	13	7,969	0.3025	0.2146–0.3905	~98	→ Stable
Oliveira et al. (24)	13	7,907	0.3062	0.2192–0.3932	~98	→ Stable
Ramadan et al. (18)	13	7,826	0.3019	0.2136–0.3901	~98	→ Stable
Jowshan et al. (34)	13	8,010	0.2762	0.2014–0.3510	~98	**↓Notable decrease**
López González et al. (30)	13	3,369	0.3150	0.2336–0.3963	~96–97	**↑Notable increase**
Bajaber et al. (32)	13	7,807	0.3000	0.2116–0.3885	~98	→ Stable
Hasan et al. (33)	13	7,978	0.2913	0.2045–0.3780	~98	→ Stable
Ayogu et al. (29)	13	8,064	0.3031	0.2159–0.3902	~98	→ Stable
Joubert et al. (17)	13	7,949	0.2763	0.2011–0.3515	97.9	**↓ Notable decrease**
Estimated global	14	8,107	0.2984	0.2166–0.3802	98.3	—

Each row shows the random-effects REML estimate after excluding one study at a time. *k*: number of remaining studies; N: total sample size of the remaining studies; ES: effect size (proportion); CI: confidence interval; I^2^: inconsistency index. Values in bold indicate influential studies whose exclusion produced the largest changes in the pooled prevalence estimate: Akintunde and Oloyede (21), Jowshan et al. (34), Joubert et al. (17), and López González et al. (30).

## 4 Discussion

### 4.1 Main findings

To our knowledge, this systematic review and meta-analysis is the first to quantify the pooled prevalence of MetS among schoolteachers. The findings indicate a substantial cardiometabolic burden in the populations studied, with a pooled prevalence of approximately one-third of participants. This estimate is broadly consistent with the upper range reported for the global adult population and for working-age populations, suggesting that MetS represents an important occupational health concern among schoolteachers (3, 4, 6). However, the marked between-study variability indicates that the pooled estimate should be interpreted as an average across diverse populations and settings rather than as a uniform prevalence applicable to all schoolteachers.

Central obesity was the dominant component of MetS in virtually all included studies, followed by dyslipidemia and hypertension. This profile resembles findings from meta-analyses of MetS in other middle-income country contexts (35, 36) and is consistent with reports on chronic non-communicable diseases among teachers (37, 38). The predominance of female participants in most studies further contextualizes this finding, given that the menopausal transition during midlife is associated with unfavorable changes in regional fat distribution, particularly increases in visceral and android fat, which may contribute to greater central adiposity (39).

### 4.2 Geographic variation as the main moderator

The subgroup analysis by geographic region was the only one that identified a statistically significant moderator (*p* < 0.001). Asian studies collectively reported the highest pooled prevalence (35%), driven in part by the particularly high prevalence in Iran ((34); 60%), as well as by consistently high rates in India, Indonesia, Malaysia, and Saudi Arabia. The higher pooled prevalence observed in Asian studies may result from the interaction of demographic, metabolic, behavioral, and occupational factors. Rapid urbanization, industrialization, and nutritional transition in several Asian countries have been associated with increased consumption of energy-dense foods, reduced physical activity, more sedentary lifestyles, and greater psychosocial stress, all of which may contribute to the growing burden of MetS in the region (36). Additionally, Asian populations may develop cardiometabolic abnormalities at lower BMI and waist circumference levels, leading to the adoption of ethnicity-specific cut-offs in the IDF and JIS definitions (36, 40). Among schoolteachers, these contextual factors may be compounded by high occupational demands, administrative workload, psychosocial stress, prolonged sedentary time, insufficient sleep, and limited opportunities for regular physical activity. Supporting this interpretation, a recent study among female teachers in urban Bangladesh reported high frequencies of insufficient physical activity, prolonged sedentary behavior, short sleep duration, moderate-to-high perceived stress, overweight or obesity, central obesity, and other cardiometabolic abnormalities (41). However, these factors should be considered plausible contributors rather than established determinants, as occupational pressure, sleep, and stress were not consistently assessed across the studies included in the meta-analysis.

The single European study (30), conducted in Spain with the largest sample size (*n* = 4,738), reported the lowest prevalence (9%). This estimate may reflect differences in population characteristics, lifestyle patterns, sampling methods, diagnostic context, and potentially protective dietary patterns; however, it should not be interpreted as representative of Europe as a whole. African studies presented intermediate rates (29%), with notable intracontinental heterogeneity: while two South African studies reported prevalences close to 29% (17, 20), the Nigerian study by Akintunde and Oloyede (21) reported only 7.2% when using harmonized criteria. In this regard, the discrepancies reported by geography are likely due to differences in population characteristics, urbanization, and the sampling strategies and diagnostic practices. Differences in healthcare access, preventive screening practices, and the detection and management of cardiometabolic risk factors may also contribute to regional variation. However, these contextual factors were not systematically reported in the included studies and could not be directly evaluated. Despite the statistical significance of geographical heterogeneity between subgroups, the very high residual I^2^ (> 95%) in Asia and Africa indicates that geographical origin alone does not fully capture the complexity of variability between studies.

### 4.3 Role of diagnostic criteria

Contrary to the usual assumption, the choice of diagnostic criterion (NCEP ATP-III, `, or JIS) did not significantly explain the observed heterogeneity (p ≈ 0.99; R^2^ = 0%). While the IDF criteria, which have lower waist circumference cut-off points, could generate higher prevalence estimates, meta-regression confirmed that no single criterion offers a systematic advantage in this specific sample of teachers. This may reflect the relatively homogeneous cardiometabolic risk profile of the included populations, in which multiple components are elevated simultaneously, regardless of the specific criterion applied. Nevertheless, methodological standardization remains a priority: the use of population-specific waist circumference cut-off points (as in the JIS criteria) may be particularly relevant for Asian populations, where cardiometabolic risk accumulates with lower body mass index values (36, 40).

### 4.4 Occupational factors, lifestyle, and quality of life

A significant limitation of the available evidence is the limited integration of occupational variables and their potential impact on health and quality of life. Schoolteachers may be particularly exposed to a combination of occupational conditions that may contribute to cardiometabolic risk (18, 19). Teaching frequently involves high psychological demands, classroom management responsibilities, administrative duties, work outside formal school hours, and limited recovery time. These demands may contribute to chronic stress, emotional exhaustion, and burnout, while reducing the time and motivation available for physical activity, healthy food preparation, and adequate sleep (11, 42). Although teaching includes periods of standing and movement, lesson planning, grading, meetings, online activities, and administrative work may generate substantial sedentary time and prolonged screen exposure (13, 20). From a psychosocial perspective, high workload, low decision latitude, limited organizational support, job insecurity, and work–family conflict may further influence health through physiological stress pathways and adverse health behaviors (43–45). However, these occupational exposures were assessed inconsistently across the included studies, preventing a quantitative evaluation of their contribution to MetS prevalence. Only one study specifically assessed work-related stress (23), whereas another analyzed physical activity using objective, device-based measurements (17). Joubert et al. (17) reported that each additional hour per week of moderate-to-vigorous physical activity was associated with a 27% lower likelihood of MetS, providing one of the most robust occupational health findings among the included studies.

In this context, insufficient physical activity is a well-established modifiable contributor to cardiometabolic risk (46). Among schoolteachers, this risk may be reinforced by prolonged static postures, sedentary administrative work, limited access to opportunities or infrastructure for physical activity, and heavy workloads that reduce the time available for regular exercise (11, 47). The magnitude of MetS among schoolteachers can also be contextualized against estimates reported in other working populations. In a cross-sectional study of 259,014 Spanish workers, Sánchez-Chaparro et al. (48) reported an overall prevalence of 9.5% using ATP III criteria, although the relatively young mean age of the sample should be considered when comparing these findings with schoolteachers. In the United States, prevalence varied substantially across occupational categories, ranging from approximately 9% among creative and technical professionals to nearly 30% among agricultural and food-service workers (49), while prevalence estimates close to 30% have been reported among German office workers (50). By comparison, the present meta-analysis found a pooled MetS prevalence of 30% among primary and secondary schoolteachers. This estimate was higher than that reported in the large Spanish working population and was comparable to the upper range observed in several sedentary or high-demand occupational groups. A meta-analysis of armed forces personnel reported a substantially lower prevalence among military personnel (8.3%), whereas the prevalence among police officers was 26.2%, illustrating important variability even within physically demanding and highly regulated occupations (51). Overall, these comparisons suggest that the cardiometabolic burden among schoolteachers is substantial and broadly comparable to that reported in some sedentary and high-demand occupational groups. However, direct comparisons should be interpreted with caution due to differences in age, sex distribution, geographic setting, occupational selection, and MetS diagnostic criteria.

In relation to these consequences, the study by Lizana et al. (37) among rural teachers in Chile, although not included in the meta-analysis because it did not report MetS prevalence according to standardized diagnostic criteria, provides relevant evidence on the potential effects of clustered cardiometabolic conditions on teachers’ quality of life. This study found that the simultaneous coexistence of two or three chronic conditions (abdominal obesity, obesity by fat mass, and hypertension) significantly increased the risk of decline in the mental health component of quality of life, although the magnitude of this estimate should be interpreted cautiously because of its statistical uncertainty. In addition, teachers younger than 45 years showed a higher likelihood of deterioration in the mental component of quality of life, suggesting that the effects of chronic cardiometabolic conditions may not be restricted to older age groups (37). This finding is particularly relevant in the context of this meta-analysis because the mean age of participants in the included studies exceeded 40 years in almost all cases, and a substantial proportion of the teaching populations analyzed were within an age range associated with increasing cardiometabolic risk and potential consequences for psychological well-being and professional performance (37, 38). Future studies should systematically incorporate validated measures of occupational exposure (teaching hours per week, class size, psychosocial stress, and work-life balance indicators) to support more robust analyses of the contribution of occupational factors to MetS risk among schoolteachers.

### 4.5 Heterogeneity across studies

The substantial heterogeneity observed in the pooled analysis (*I*^2^ = 98.34%) reflects the combined influence of differences in diagnostic definitions, population characteristics, and methodological approaches across studies. From a public health perspective, the included studies used different definitions of metabolic syndrome, primarily those proposed by the International Diabetes Federation, the NCEP ATP III, and the Joint Interim Statement. These definitions differ in waist circumference and glucose thresholds, in whether central obesity is required for diagnosis, and in how pharmacological treatment is incorporated into individual MetS components (40, 52, 53). Such differences may alter participant classification and, consequently, prevalence estimates. Nevertheless, the subgroup estimates were similar across the IDF, NCEP ATP III, and Joint Interim Statement criteria. This convergence may be explained by the substantial overlap among their core components, as all three definitions assess central obesity, elevated blood pressure, hyperglycemia, hypertriglyceridemia, and reduced HDL cholesterol. Although they differ in specific thresholds and in whether central obesity is mandatory, individuals with multiple cardiometabolic abnormalities are likely to meet the diagnostic requirements under more than one definition. In addition, the influence of diagnostic criteria may have been smaller than that of broader differences in age, sex distribution, nutritional status, geographic setting, and baseline cardiometabolic risk across the included populations. However, these subgroup comparisons should be interpreted with caution because the number of studies in some diagnostic categories was limited. Study populations also varied in age, sex distribution, nutritional status, ethnicity, geographic region, and baseline cardiometabolic risk, all of which are closely related to the prevalence of MetS (3, 6). Methodologically, studies differed in sampling strategies, sample size, representativeness, data collection period, and methodological quality. Additionally, potentially relevant occupational exposures, including working hours, sedentary time, psychosocial stress, physical activity, and employment conditions, were reported inconsistently and could not be quantitatively evaluated (11, 16, 54, 55). Although subgroup analyses were conducted according to sex, geographic region, diagnostic criteria, and methodological quality, substantial residual heterogeneity remained, indicating that the observed variability was multifactorial and partly attributable to unmeasured study- and population-level characteristics. In this context, the pooled prevalence should be interpreted as an average estimate across different occupational and epidemiological settings rather than as a single prevalence uniformly applicable to all schoolteacher populations (28, 56).

### 4.6 Implications for public and occupational health

The findings have practical implications in both clinical practice and health policy. Teachers constitute a strategically important workforce whose cardiometabolic health directly influences absenteeism, productivity, early retirement, and, critically, the quality of education students receive (13, 14). Workplace-based cardiometabolic screening may facilitate the early identification of MetS and support timely preventive action in school settings. Interventions combining structured physical activity programs, nutrition education, and stress management may contribute to improving cardiometabolic health in school settings. In particular, nutrition education programs implemented among schoolteachers have been shown to improve knowledge, attitudes, and practices related to balanced nutrition (57, 58).

From a sex-disaggregated perspective, prevention programs should consider the predominantly female composition of the teaching workforce, with particular attention to women undergoing the menopausal transition. This period is associated with unfavorable hormonal and metabolic changes, including increased visceral and android adiposity, impaired insulin sensitivity, and a higher incidence of metabolic syndrome, which may increase cardiometabolic vulnerability in midlife women (39, 59). Schools should be conceptualized not only as environments for promoting children’s health, but also as workplaces that require structured occupational health frameworks (15).

### 4.7 Limitations and strengths

The main limitations of this study were as follows: (1) all included studies were cross-sectional, which precluded causal inference; (2) very high residual heterogeneity (*I*^2^ = 98.3%) persisted even after subgroup analyses; (3) most studies had limited control of confounding factors; (4) occupational variables were insufficiently assessed in the included studies; (5) the assessment of publication bias was limited by the small number of included studies, which reduced the statistical power of Egger’s test and precluded definitive conclusions regarding funnel plot asymmetry; and (6) Europe and South America were represented by only one study each, limiting regional generalizability. Nevertheless, the study had several methodological strengths: (1) to our knowledge, it was the first meta-analysis to provide a pooled estimate of MetS prevalence among schoolteachers; (2) it followed the PRISMA 2020 guidelines and was prospectively registered in PROSPERO; (3) it applied a standardized quality assessment instrument (NHLBI) across all included studies; (4) it performed subgroup analyses according to sex, methodological quality, diagnostic criteria, and geographic region, together with a leave-one-out sensitivity analysis; and (5) it formally assessed publication bias.

## 5 Conclusion

This systematic review and meta-analysis indicates that MetS represents a substantial occupational health concern among primary and secondary school teachers, affecting approximately three in ten individuals across the populations studied. However, due to the extremely high heterogeneity observed among the studies, this result should be interpreted as an overall average estimate rather than a uniform prevalence applicable to all teaching contexts. Among the components of MetS, central obesity was the most frequently reported, followed by dyslipidemia and hypertension, suggesting a cardiometabolic profile predominantly associated with abdominal adiposity and metabolic abnormalities.

From a public health and occupational medicine perspective, these findings support the development of cardiometabolic screening initiatives and workplace-based lifestyle interventions adapted to the characteristics of the teaching profession. Future research should prioritize longitudinal designs, standardized diagnostic criteria with population-appropriate thresholds, objective measurement of physical activity, and systematic assessment of occupational exposures, including job stress, teaching workload, and socioeconomic conditions. Stratified analyses by sex and age would further improve the applicability of the findings to the design of prevention strategies focused on educational settings.

## Data Availability

The original contributions presented in the study are included in the article/supplementary material, further inquiries can be directed to the corresponding author.
